# Exploring Medical Information Needs and Accessibility in Swedish Dental Care by Analysis of Documentation Workflows and Electronic Dental Records in Dalarna: Sociotechnical Qualitative Study

**DOI:** 10.2196/82691

**Published:** 2026-01-29

**Authors:** Sahid Hasan Rahim, Nadia Davoody, Stefano Bonacina

**Affiliations:** 1 Health Informatics Centre, Department of Learning, Informatics, Management and Ethics, Karolinska Institutet, Tomtebodavägen 18 A, Stockholm, 17177, Sweden, +46 8 524 83346

**Keywords:** dental informatics, dental care, health information exchange, health information interoperability, health information systems, medical informatics

## Abstract

**Background:**

Despite growing evidence demonstrating the connection between oral and systemic health, medical and dental care remain institutionally divided. A significant consequence of this division is the lack of information sharing, which is particularly problematic in dental care, where knowing patients’ medical information is crucial for providing safe and effective treatments. This separation poses additional challenges in Swedish regions with limited resources, such as Dalarna, where dental care practices would benefit from improved access to relevant medical information in their electronic dental record (EDR) systems.

**Objective:**

This study aimed to explore how current documentation workflows and EDR systems support the medical information needs within dental care practices in Dalarna and consider what influence direct access to medical information could have.

**Methods:**

The study adopted an exploratory-descriptive qualitative approach. Semi-structured interviews were conducted with dental practitioners working in general dental practices. Data collection followed a sociotechnical framework, and thematic analysis was performed to identify key medical information needs, as well as current workflow and system limitations. Conceptual models were developed to reflect these findings.

**Results:**

Eighteen dental practitioners were interviewed. The identified medical information needs included specific types of medical conditions, pharmacological information, treatment history, and laboratory values. Furthermore, dental practitioners highlighted substantial challenges in existing documentation workflows and the EDR system. Proposed conceptual models demonstrated how integrating EDR systems with the Swedish National Patient Overview (“Nationell Patientöversikt”) via National Service Platform (“Nationell Tjänsteplattform”) could streamline workflows and enhance information accessibility.

**Conclusions:**

The findings show a clear need to improve medical information accessibility in dental care. A solution is to facilitate interoperability and align digital infrastructure with the identified needs. The proposed recommendations offer a feasible starting point for improving medical information access in Swedish dental care, particularly in resource-constrained regions like Dalarna.

## Introduction

### Medical Information in Dental Care

Comprehensive medical information is essential in dental care, as inaccurate or incomplete patient medical history assessments can lead to adverse treatment outcomes [1]. In addition to patient safety, the absence of accurate medical information exposes dental professionals to potential harm, as many procedures are invasive, carrying risks of occupational hazards such as cross-contamination and disease transmission [2]. The literature highlights several types of medical information as particularly relevant to dental care. Medical history, especially patients’ diagnoses and current medications, is consistently prioritized [3-5]. Information about allergies and adverse drug reactions is also essential, as overlooking these may lead to severe or even life-threatening scenarios [3,4,6]. Additionally, details such as medical treatment plans, hospitalization history, and surgical history are considered highly valuable, as they support improved care coordination and more effective treatment planning [3-5]. Together, these categories form the foundation of the medical information that dental professionals rely on to deliver care. Unfortunately, challenges arise due to inconsistencies in the documentation and communication of such information, particularly as it is often self-reported by patients [7]. This raises the question of whether health information technology can possibly address this gap and support more reliable access to critical medical information in dental care.

### Information Technology for Record-Keeping

In dental care, patient information is primarily managed through electronic dental records (EDRs), whereas medical care relies on electronic health records (EHRs) [8-10]. The advent of such information systems has facilitated the process of recording patient information in data format, thereby giving professionals in both dental and medical care a more structured and streamlined approach to record-keeping [9,10]. This also includes the process of enabling information exchange.

### Interoperability

To enable the exchange of patient information between EHR and EDR systems, interoperability is required [11]. Interoperability refers to the ability of different systems to exchange, process, and use patient data effectively, and it is commonly described across four levels. The technical level involves a shared technical infrastructure that allows systems to communicate. The syntactic level requires data to be structured in a standardized format for processing. The semantic level ensures that data are interpretable and meaningful across systems. Finally, the organizational level emphasizes that interoperability at the technical, syntactic, and semantic levels depends on alignment with regulatory, administrative, and policy frameworks, including agreements that enable data exchange while ensuring compliance with laws and regulations.

### Current Dental-Medical Interoperability Efforts

The literature identifies two main approaches to achieving interoperability between dental and medical care systems. Health information exchange (HIE) serves as an intermediary to facilitate data exchange between different systems [3,12-14]. Integrated EHR-EDR (IEHR), on the other hand, operates on a single-system architecture [14]. This system has become widely adopted by organizations offering integrated dental-medical care services [15].

While both IEHR and HIE aim to facilitate information exchange between dental and medical care, their feasibility depends on organizational size, infrastructure, and financial resources. IEHR can provide data conformity and reliability, but may be financially and technically restrictive for smaller organizations [4,7,14]. In contrast, HIE can offer a more flexible and cost-effective solution, though usability barriers may impede its adoption [3,12-14].

Understanding how these approaches are implemented within a specific national context is essential for assessing their real-world applicability. Here, Sweden offers a particularly instructive case due to its publicly funded health care system, high digital maturity, and ongoing efforts to advance HIE [16,17]. In fact, Sweden’s broader aim is to become a global leader in health care digitalization by 2025, a vision that includes both medical and dental care [18].

### The Swedish Interoperability Strategy

Sweden is a country divided into 21 administrative regions, each governing its publicly funded medical and dental care services autonomously [19]. Due to this decentralized administrative structure, challenges have arisen in ensuring the accessibility and exchange of patient data across regions and organizations [17,20]. Hence, the Swedish government enacted the Integrated Health and Social Care Documentation Act (2022:913), establishing a legal basis for the sharing of patient information across health care services [21]. This legal foundation was followed by the implementation of nationwide digital infrastructure, the national patient overview (nationell patientöversikt; NPÖ), and the national service platform (nationell tjänsteplattform; NTjP) [22-24]. NPÖ is a nationwide digital platform primarily provided to public health care providers, allowing authorized health care professionals to retrieve patient data across organizations and regions [23]. NTjP refers to the technical infrastructure that facilitates the exchange of patient data in a standardized and secure manner [22,24]. This concept is similar to HIE, as it acts as an intermediary layer between different systems in organizations and regions.

Currently, the digital infrastructure does not support information exchange between dental and medical care services [25]. It may pose particular challenges in regions where access to both services is already constrained, particularly in underserved or rural regions [26-29]. To illustrate the relevance and urgency of such an approach, the region of Dalarna provides a meaningful context for further discussion.

### The Dalarna Region

One of the most pressing issues in Dalarna is the shortage of dental professionals, with the lowest dentist-to-population ratio in Sweden [30]. Due to its vast land area, patients in remote regions may need to travel long distances to reach a general dental practice (GDP). In addition, Dalarna has an aging population, with nearly 25% of residents aged 65 years or older, compared to the national average of 19% [31]. This demographic trend places further strain on an already limited dental workforce, as older adults often require more frequent and complex dental and medical care [32]. Facilitating access to medical information in dental care in Dalarna could therefore support more effective and higher-quality care outcomes for patients.

### Problem Description

Sweden’s digital infrastructure, the NPÖ and the NTjP, support information exchange between health care information systems across regions and organizations [22-24]. However, these platforms do not currently provide access to medical information in dental care [25]. This lack of integration presents a missed opportunity to enhance both patient safety and efficiency of dental workflows, especially in rural regions where access to medical and dental services is limited [26-29]. The Dalarna region exemplifies this challenge, facing one of the most severe shortages of dental professionals in the country. This shortage forces many residents to travel long distances for dental care [30]. Yet, addressing this gap does not necessarily require extensive structural or financial reforms. Rather, a pragmatic path forward may lie in leveraging existing infrastructure to enable dental professionals to access relevant medical information directly through their EDR systems. However, without a clear understanding of what specific medical information is needed and how it fits into dental workflows, such interoperability efforts risk being ineffective or even disruptive. Although existing literature reports the types of medical information relevant to dental care [3-5], it lacks specificity. Therefore, it is crucial to identify the specific medical information needs of dental professionals and examine how these can be supported within current workflows, systems, and organizational structures. This necessitates a sociotechnical approach that accounts for both technical components and end-users [33,34], primarily dental professionals.

### Aim

This study aims to explore how current documentation workflows and EDR systems in dental care practices in the Dalarna region in Sweden support or limit the fulfillment of the medical information needs, and to examine the potential impact of having direct access to accurate and relevant medical information within EDR systems.

## Methods

### Research Design

This study adopted an exploratory-descriptive qualitative research approach to allow for a comprehensive yet structured and contextualized examination of the experiences, challenges, and needs on the subject matter [35].

### Study Setting

The study was decided to be conducted in GDPs operated under the Public Dental Service Dalarna (PDSD), which is administered by the regional authority Region Dalarna and is responsible for providing dental care services within the region [36]. Consequently, private dental clinics or organizations and specialist clinics were excluded.

### Participants and Sampling

The primary participants consisted of dental practitioners, as they are directly responsible for patient care [37]. Only dental practitioners having their main professional duties within GDPs operated by PDSD were considered. Purposive sampling was used to identify participants who were able to provide detailed, precise, and reliable information aligned with the study’s aim [38]. Conversely, other dental professionals were excluded.

### Data Collection

Interviews were deemed the most appropriate data collection method as they offered an opportunity for participants to share their perspectives in-depth related to the subject matter [39]. The interviews followed a semi-structured format to ensure a balance between systematic inquiry and flexibility. The interviews needed to capture the sociotechnical aspects relevant to the subject matter. To achieve this, an interview guide was designed following a sociotechnical framework. The sociotechnical framework defined by Sittig et al [40] provides a comprehensive lens for evaluating health information technology by examining the interaction between information systems and the social context in which they operate. It comprises 8 dimensions, where for this study, 6 dimensions were chosen based on their alignment with the research aim [40]: (1) clinical content to identify the specific medical information considered important and what should be accessible in EDR systems; (2) people to explore experiences and challenges in obtaining medical information from patients, the medical domain, or other sources; (3) human-computer interface to assess the usability of the current EDR system regarding accessibility and documentation of medical information; (4) workflow and communication to understand how medical information is obtained and documented within current workflows, focusing on the patient and EDR system; (5) internal organizational policies, procedures, and culture to examine existing policies on documentation and retrieval of medical information within PDSD; and (6) external rules, regulations, and pressures to explore the awareness of guidelines or requirements for documenting medical information in dental care. These dimensions informed the development of the interview guide in Swedish and English (Multimedia Appendix 1 ).

### Assessment and Pilot Testing

Following development, the interview guide was reviewed by the co-authors, SB, ND, and the Head of Division at PDSD, to confirm its suitability. The guide was subsequently pilot-tested, which confirmed that the questions elicited comprehensive responses. Individuals involved in the review and pilot testing were excluded from the final study to avoid diluting the results. A bilingual consent form in both Swedish and English was developed in parallel (Multimedia Appendix 2).

### Recruitment and Interview Sessions

The head of division at PDSD facilitated recruitment by distributing interview invitations to GDPs. Interviews were conducted digitally via Microsoft Teams between March 7 and July 28, 2025. Digital interviews were chosen to accommodate participants from various locations and minimize travel-related costs. All interviews were audio-recorded and transcribed using Microsoft Teams’ built-in tool. Supplementary notes were taken during each session to capture contextual and paralinguistic cues. Transcripts were carefully reviewed, compiled, and returned to participants to confirm accuracy. Interviewing concluded when data saturation was reached, meaning no new information emerged, and the data were sufficiently rich to address the study’s research questions [41].

### Data Analysis

#### Thematic Analysis

Thematic analysis was conducted to identify and interpret patterns within the interview data, guided by the sociotechnical framework from Sittig et al [40]. The analysis followed Braun and Clarke’s 6-phase approach [42]. Transcripts were read repeatedly to gain an in-depth understanding of the data, with audio recordings revisited when meaning was unclear. Excerpts were then collated into potential themes, which were reviewed and refined to ensure they accurately represented the data. Themes were organized under the following columns: sociotechnical dimension, participant (anonymized), excerpt, subtheme, and theme. The entire analysis was iterative, with frequent revisions of subthemes and themes to ensure a valid and coherent representation of the findings [43].

#### Conceptual Modeling

Following the analysis, conceptual models were developed to illustrate current and proposed workflows for accessing medical information in dental care. These models considered integration with existing Swedish infrastructures. Unified Modeling Language (UML) was used due to its use in health system planning and stakeholder communication [44,45]. Three UML diagrams were created. Activity diagrams were used to show current and improved workflows for obtaining and documenting medical information in dental care. Use case diagrams were developed to present expected system functionalities derived from the interview data. Finally, a sequence diagram was created to illustrate how medical information could be exchanged from regional or national infrastructures to local EDR systems.

### Ethical Considerations

The study was carried out in Sweden. According to the Swedish Ethical Review Act (SFS 2003:460) [46] and guidance from the Swedish Ethical Review Authority [47], the type of research presented in this article does not require formal ethical approval, as it does not involve sensitive personal data as defined by the European Union General Data Protection Regulation (EU 2016/679) [48]. Nonetheless, we emphasize that all ethical standards were strictly followed in accordance with relevant legislation, the Declaration of Helsinki [49]. Participants were fully informed about the study’s purpose, their rights, and data handling procedures. Informed consent was obtained both in writing and verbally. Participation was voluntary, and withdrawal was permitted at any time. Anonymity was guaranteed, and participants were informed that findings would be publicly disseminated.

## Results

### Descriptive Overview of the Interview Sample

In total, 18 interviews were conducted, after which no new information emerged beyond the 11th interview. All participants reported using the T4 EDR system, developed by Carestream [50]. Fifteen participants identified as female and three as male. Key characteristics of the participants are presented in Table 1. All participants treated diverse groups of patients and performed a broad range of general dental treatments, where 8 participants identified oral surgery as their main focus. All participants primarily used free text.

**Table 1.** Key characteristics of the participants.

Participants	Age (year range)	Work experience overall (year range)	Work experience Dalarna (year range)	Scope of practice
P1	40‐45	10‐15	10‐15	General Dentistry and Oral Surgery
P2	25‐30	0‐5	0‐5	General Dentistry
P3	25‐30	0‐5	0‐5	General Dentistry
P4	25‐30	0‐5	0‐5	General Dentistry, Pediatric Dentistry, and Orthodontics
P5	25‐30	0‐5	0‐5	General Dentistry and Oral Surgery
P6	25‐30	0‐5	0‐5	General Dentistry
P7	45‐50	20‐25	10‐15	General Dentistry, Oral Surgery, Pediatric Dentistry, and Orthodontics
P8	25‐30	0‐5	0‐5	General Dentistry, Oral Surgery, and Prosthodontics
P9	45‐50	25‐30	5‐10	General Dentistry and Oral Surgery
P10	25‐30	0‐5	0‐5	General Dentistry and Oral Surgery
P11	25‐30	0‐5	0‐5	General Dentistry, Oral Surgery, and Pediatric Dentistry
P12	20‐25	0‐5	0‐5	General Dentistry
P13	25‐30	0‐5	0‐5	General Dentistry
P14	35‐40	5‐10	0‐5	General Dentistry, Oral Surgery, and Prosthodontics
P15	35‐40	0‐5	0‐5	General Dentistry
P16	25‐30	0‐5	0‐5	General Dentistry
P17	35‐40	5‐10	5‐10	General Dentistry and Community Dentistry
P18	25‐30	0‐5	0‐5	General Dentistry

### The Specific Medical Information Needs – Clinical Content

Under the sociotechnical dimension of “clinical content*,”* the interview data revealed three overarching categories of medical information needs: “conditions, pharmacological information, treatment history and laboratory values*.”* Each category contains specific types of information that participants deemed essential for providing dental care (Table 2).

**Table 2.** Specific medical information identified within the sociotechnical dimension “clinical content.”

Themes		Participants
Conditions		
Oncological diagnosis		P3, P5, P7, P8, P9, P11, P12, P13, P15, P16, P17, P18
Psychiatric and neurological disorders		P2, P4, P7, P11, P12, P14, P18
Cardiovascular diseases		P2, P3, P5, P8, P13, P14, P15, P16, P17, P18
Hematologic disorder		P1, P15, P17
Hepatic diseases		P3
Pulmonary diseases		P3, P17
Renal diseases		P3, P17, P18
Rheumatism		P3
Diabetes		P7, P14
Sleep apnea		P9
Infectious disease		P11, P17
Allergies in general		P2, P12, P14, P15, P18
Pharmacological information		
Penicillin allergy		P11, P14, P16
Substance abuse		P11
Comprehensive medication list		P1, P3, P4, P6, P10, P12, P15
Anticoagulants		P1, P2, P5, P6, P7, P8, P13, P14, P16, P17, P18
Antiresorptive agents		P1, P2, P3, P6, P7, P8, P9, P10, P11, P12, P13, P14, P16, P17, P18
Psychiatric medications		P2, P4, P11, P12, P16, P17, P18
Treatment history and laboratory values		
Ongoing oncological treatment		P5, P7, P8, P9, P11, P12, P16, P17
Surgical procedures		P1, P5, P10, P13, P18
Blood coagulation marker		P8, P11, P14

### Conditions

Among conditions, oncological diagnoses and cardiovascular diseases were the most frequently emphasized because of the associated risks when providing invasive treatments. For patients with oncological diagnoses, participants highlighted that treatment planning and risk management are directly influenced by the potential effects of cancer on oral healing and immune response. Similarly, participants reported that patients with cardiovascular diseases require special attention due to systemic risks associated with invasive procedures:

*Knowing if a patient has cancer or has had it in the past can influence the treatment we provide in terms of healing outcomes […] healing can be delayed, and there is a greater risk for them to get infected.*

[P9]

*To be aware of patients with cardiovascular diseases, where the choice of anesthesia matters. There is also a bleeding risk, and some may need antibiotic prophylaxis.*

[P14]

Psychiatric and neurological disorders were also commonly mentioned due to their impact on patient cooperation and communication:

*Neurodivergent conditions matter, particularly when working with children. It influences how you communicate and treat them.*

[P4]

Allergies in general were also deemed important, as considerations need to be taken in relation to the materials used in dental care:

*Some patients have sensitivities to certain materials, so we need to be careful. This is particularly important to know in [dental] prosthetic treatments, where you are dealing with crowns or removable dentures.*

[P14]

Additionally, infectious diseases were highlighted due to their relevance in maintaining a safe clinical environment for both patients and dental professionals:

*Being aware of any infectious diseases is important, not only for our own safety but also to prevent cross-contamination between patients.*

[P11]

Other conditions were brought up by individual participants. Though each was mentioned less frequently, they reflect the complexity of the oral-systemic health correlation in the context of coordinating dental care:

*Patients often have several different diagnoses, such as heart problems, kidney issues, liver disease, or lung conditions, and each of those can have a real impact on oral health. Sometimes it is not clear how it all is related [for the patient], but it definitely affects how we manage them chairside.*

[P3]

*For patients with diabetes, wound healing is affected, and there is a higher risk of infection and periodontal disease.*

[P14]

### Pharmacological Information

The pharmacological information theme comprises medications that participants regarded as critical, primarily due to the procedural risks associated with specific drugs. The most frequently mentioned medication category was antiresorptive agents and anticoagulants due to complications associated with dental treatments:

*If we miss that someone has been on multiple blood thinners or bisphosphonates, and they do not mention it because they’re no longer taking them or do not think it is important [to mention], it can lead to problems like excessive bleeding or delayed wound healing.*

[P13]

Additionally, several participants expressed the importance of knowing whether patients are taking psychiatric medications*,* since that would increase their understanding of patient behavior and anxiety-induced responses during care:

*I would actually like to see more information about psychiatric medications and prescriptions. I feel like we often miss that type of medication. We could better tailor our care for these patients, particularly in cases where these medications, combined with anesthetics or sedatives, affect how a patient responds to treatment, like increased anxiety.*

[P2]

Penicillin allergies and substance abuse were also mentioned, as there were concerns about prescribing antibiotics or analgesics, particularly due to risks of adverse reactions or potential misuse by patients:

*Someone who lived in another region started coming to clinics here in Dalarna asking for morphine. We did not know about the substance abuse at first, but then we received a warning from the local pharmacy saying we should not prescribe anything to her. […] If the patient is allergic to penicillin, we need to know so we do not prescribe it.*

[P11]

### Treatment History and Laboratory Values

In treatment history and laboratory values*,* participants frequently considered ongoing oncological treatment as essential information for treatment planning and to prevent complications:

*If they [patients] undergo radiation therapy [for cancer], it is really important that we know about it. Not just that they had treatment, but also which region was irradiated. That kind of detail affects how we approach even routine dental procedures.*

[P9]

Surgical procedures were also referenced since such information is used to assess potential risks and determine whether treatment should be delayed:

*If someone has had an operation, we need to know, because they may have to receive antibiotic prophylaxis. I may also decide to postpone treatments.*

[P18]

Likewise, information on blood coagulation markers was commonly sought to support decisions around the timing of treatments for patients and to evaluate their bleeding risks:

*For patients on warfarin, having access to their International Normalized Ratio value is essential. It helps me determine whether I can proceed with treatment immediately or if I need to postpone or take extra precautions.*

[P8]

### Current Workflow: People

The sociotechnical dimension of people focused on identifying who the participants interact with in relation to obtaining medical information. As summarized in Table 3, it revealed that medical information is predominantly derived from patient reporting, proxy reporting, or through medical consultation. In general, the findings demonstrated how human factors influence the workflow, suggesting the need for medical information to be made more accessible.

**Table 3.** Sources for obtaining medical information, categorized under the sociotechnical dimension of “People”.

Themes	Participants
Patient reporting	
Verbal recall	P1, P2, P3, P4, P6, P8, P9, P12, P14, P15, P16, P17, P18
Medication list	P5, P7, P8, P10, P11, P13, P15, P16, P17, P18
Online patient portal	P2, P8, P10, P11, P13, P18
Incomplete or inaccurate reporting	P1, P2, P3, P4, P5, P6, P7, P8, P9, P10, P13, P14, P15, P16, P17, P18
Proxy reporting	
Companions	P1, P2, P17
Care home	P1, P2, P5, P12
Direct relatives	P5, P11, P17
Challenges in proxy reporting	P1, P2, P11, P12
Medical consultations	
Primary care	P1, P2, P4, P5, P6, P8, P10, P11, P13, P15, P17
Hematology	P5, P7, P9, P12
Cardiology	P9, P11, P18
Oncology	P1, P9
Oral and Maxillofacial Surgery	P1, P10
Nephrology	P9
Coordination limitations	P1, P2, P4, P5, P6, P7, P8, P10, P13, P14, P15

### Patient Reporting

Participants reported that medical information was often obtained through verbal recall, medication lists*,* or by asking patients to access their online patient portal. Verbal recall relied on patients remembering their medical history; medication lists were paper documents brought in by patients; and the online patient portal was used to verify details when verbal reports were insufficient, or no list was available:

*Medical history is mostly obtained from the patients themselves verbally.*

[P4]

*Sometimes I ask the patient to log in to their online patient portal. The elderly do not know how, so I usually rely on what they say, or ask them to bring a medication list later.*

[P8]

A recurring issue was incomplete or inaccurate reporting, mainly due to patients forgetting diagnoses or medications, particularly amongst the elderly or those without a caregiver:

*Older patients may not know their diagnoses or medications. Also, some patients deliberately leave things out. Just asking is not always enough.*

[P4]

Participants also noted that patients often unintentionally omitted relevant details or assumed they had nothing to report:

*Some patients say that they do not take any medications, but then later it turns out that they take seven!*

[P3]

Some also faced barriers when patients were unwilling or unable to disclose their history due to communication breakdowns:

*Sometimes there are communication difficulties, particularly with foreign patients who may struggle with the [Swedish] language. Some patients are also unwilling to share their medical history.*

[P14]

### Proxy Reporting

There are situations where medical information is obtained through proxy reporting, which in this case refers to: direct relatives, companions, or care home personnel. Companions refer to those physically accompanying the patient. There were challenges presented here as well:

*I remember one patient who arrived with a care home worker who did not know the patient’s medical history. The patient had a neurodegenerative condition, so we were unable to obtain the information directly. We tried contacting the care home, but there was no nurse available at the time. Eventually, we received a phone number for a direct relative, who was able to provide us with the necessary information.*

[P2]

### Medical Consultations

Participants described engaging in medical consultations to obtain relevant information or seek advice on complex cases. Primary care units were the most frequent point of contact, although secondary care was also involved, depending on the patient’s condition:

*I would estimate that I contact primary care units about 1–3 times per month. Occasionally, I call specific doctors directly to confirm details if I suspect the information provided by the patient is inaccurate or incomplete.*

[P4]

*I contact medical departments several times a month. Hematology for chemotherapy patients, oncology for treatment schedules, as well as nephrology and cardiology for patient-specific matters.*

[P9]

However, participants often encountered coordination limitations, such as delayed or absent responses, which hindered decision-making and delayed treatments:

*The main issue with calling medical professionals is that they are hard to reach. You often have to call multiple times, and that takes time. This often leads to postponing treatments, even for emergency cases. I cannot proceed if I am not sure about a patient’s medical history.*

[P4]

Some reported being redirected or denied help, further complicating access to essential information:

*I once called a primary care unit, and they responded strangely. They asked how I had gotten their number. Even after I explained that I was a dental practitioner and provided the necessary patient details, they refused to share any information.*

[P5]

To cope with these challenges, some participants began asking patients to coordinate directly with their medical providers:

*I once tried to contact a pediatrician, but it was very difficult to reach them. They had many patients, and it took nearly a year to get a phone appointment. Since then, I have let the patients handle the contact themselves. […] They [patients] ask their doctor, for example, whether they need antibiotic prophylaxis or if they should stop their blood thinner before an extraction, and then they get back to us with the answer.*

[P14]

### Current Workflow: Human-Computer Interface

The sociotechnical dimension of the human-computer interface examined the interaction with the EDR system when retrieving, documenting, and managing medical information (Table 4). Overall, participants emphasized the interface’s critical role in the efficient and reliable handling of medical information, revealing both challenges and opportunities for improving usability.

**Table 4.** Aspects of current interface design and expressed design needs, categorized under the sociotechnical dimension of “human-computer interface.”

Themes	Participants
Free text input	
Medical history section	P1, P4, P8, P9, P12, P14, P15, P16
Progress notes	P2, P3
Adaptive documentation behavior	P5, P6, P7, P10, P11, P13, P17, P18
Current interface limitations	
Information reliability	P1, P3, P12, P13, P17, P18
Information visibility	P2, P7, P9, P10, P15, P17
Manual data entry	P4, P6, P11, P12, P13, P14, P16, P17, P18
Design needs	
Annotations	P5, P6, P18
Improved information overview	P6, P15, P16, P17, P18
Confirm review	P6
Cross-disciplinary substance abuse alert	P10, P11
Integrated external data accessibility	P1, P2, P3, P4, P5, P6, P7, P8, P9, P10, P11, P12, P13, P14, P15, P16, P17, P18

### Free Text Input

All participants relied solely on free text input when documenting medical information in the EDR. This was done either in the medical history section (a structured form with predefined fields) or in progress notes (where other dental care activities are recorded).

*There is a medical history section form that covers common illnesses like asthma, osteoporosis, cancer, diabetes, cardiovascular disease, liver and kidney diseases, rheumatism, mental health issues, history of radiation therapy, bleeding disorders, allergies, etc. If yes to any question, we ask them what medications they take and type it in.*

[P14]

Some participants preferred documenting directly in progress notes due to time constraints and navigational hurdles:

*In the EDR, we have a medical history section to fill in information. […] However, due to time constraints, I do not have time to go through everything, so I type in the progress notes instead.*

[P2]

Most adopted an adaptive documentation behavior, using both sections to reinforce critical information:

*I use the dedicated medical history section for documenting medical information. If there is something important, like bisphosphonates, I also type it in the progress notes.*

[P6]

### Current Interface Limitations

The category current interface limitations revealed several usability issues. One major concern was information reliability*,* where participants questioned the accuracy and completeness of previously entered medical information in the EDR system:

*The problem with the current system is that we do not know if the medical information is recorded accurately or not by previous colleagues, or even if it is reliable.*

[P1]

Another issue was information visibility, where inconsistent documentation across sections made it difficult to locate medical details:

*If medical information is buried in the progress notes, it takes time to find. Time is also wasted when it is missing or outdated.*

[P7]

Manual data entry was also a major limitation. Participants described it as time-consuming and prone to errors, especially when documenting long medication lists:

*When there is a lot of medical information, entering it manually is a burden because it takes time, which could have been spent with the patient. Also, correctly spelling the names under time pressure can lead to mistakes.*

[P17]

### Design Needs

The findings in design needs emphasized the need for better system integration, interface enhancements, and improved visibility of patient data. All participants expressed a strong preference for integrated external data accessibility, where medical information should be directly accessible within the EDR:

*Compared to EHR systems, which display all medications and diagnoses, EDR systems fall short. […] Our work is also time-pressured, which is why an integrated solution providing access to medical information would save time. […] Even if in another program, that is still better than nothing.*

[P5]

Some also emphasized the need for access control, ensuring that only authorized personnel could view sensitive data:

*Ideally, all relevant medical information should be retrieved in a controlled process to safeguard patients’ privacy.*

[P15]

Additional interface improvements suggested included:

*Annotations: If there was a built-in description of each entry, that would be incredibly helpful. Like a pop-up suggestion that says what a particular drug is for.*

[P5]

*Improved Information Overview: An overview of the [medical] information is preferable, but it must be possible to filter it, so the overview stays clear and focused.*

[P17]

*Confirm Review Feature: It would be best if there was a function where you could sign to confirm that you have reviewed the medical history. […] to ensure accountability.*

[P6]

*Substance Abuse Alerts: We had a patient […] who seeks emergency care only to obtain narcotic medications. […] In EHR systems, this patient has a warning that flags up for that reason. I would like to see such functionality integrated into the EDR.*

[P10]

### Current Workflow: Workflow and Communication

Workflow and communication focused on broader clinical activities involving interactions with both the EDR system and patients in the context of managing medical information (Table 5). Overall, the findings indicate that workflow efficiency and patient safety are strongly linked to the availability and clarity of medical information. When such information is incomplete or difficult to retrieve, it disrupts clinical routines and compromises care.

**Table 5.** Challenges and limitations of the current workflow, as well as perceived benefits of direct medical information access, categorized under the sociotechnical dimension of “workflow and communication.”

Themes	Participants
Current documentation workflow	
Time-consuming	P1, P2, P5, P6, P9, P10, P11, P13, P14, P15, P16, P17, P18
Delay treatments	P1, P2, P3, P4, P5, P6, P8, P11, P13, P16
In-treatment hazards	P1, P4, P7, P8, P13, P18
Patient frustration	P8, P10
Direct information accessibility	
Clinical time management	P1, P2, P4, P5, P7, P8, P9, P10, P11, P12, P13, P14, P16, P17, P18
Improved treatment planning	P3, P7, P8, P17
Cognitive load	P3, P12
Better decision-making	P4, P6, P7, P12, P13, P14, P15, P17, P18

### Current Documentation Workflow

Participants overwhelmingly described the current documentation workflow as time-consuming, marked by frequent disruptions when obtaining and recording medical information:

*Asking patients about their medical history, then reading and documenting it accurately, takes up valuable time from the appointment.*

[P18]

These issues often led participants to delay treatments*,* which often involved canceling or rescheduling appointments:

*“Sometimes, I cannot proceed with the treatment if I do not have the patient’s full medical history. I have to explain to the patient that I cannot do anything at that moment because it would pose a risk to their health.*

[P2]

In some instances, essential medical information would emerge during procedures, posing potential in-treatment hazards**:**

*One time, a patient told me he was healthy, and just before the [tooth] extraction, he casually mentioned […] a serious condition. […] We had to stop the procedure immediately.*

[P7]

Finally, repeated appointments caused by information gaps contributed to patient frustration:

*I had to postpone the treatment […] The coordination turned out to be difficult, and the patient became increasingly frustrated.*

[P8]

### Direct Information Accessibility

Having direct information accessibility was consistently identified as a key improvement. The most frequently perceived benefit was enhanced clinical time management, as direct access would reduce administrative tasks and allow more focus on patient care:

*“It would be incredibly helpful to have access to patients’ full medical history. Then, I would not need to ask the patients because I would be able to look it up myself directly. That way, I could plan treatments faster instead of calling around for additional information.*

[P2]

Others highlighted how improved treatment planning would be possible by enabling better scheduling and avoiding unnecessary visits:

*If I had direct access, I would be able to plan better. Like, if a patient takes a certain medication, I would know that they need to come within 6 months before their next dose.*

[P3]

Also, better decision-making was noted as a distinct benefit. Several participants stressed that it would reduce uncertainty, improve efficiency, and enhance patient safety:

*Having direct access to medical information would speed up decision-making and let patients get treated faster. I would also feel more secure making the right call.*

[P6]

A final point was the reduction of patients’ cognitive load, as they would no longer need to recall or bring documentation themselves:

*Patients would not have to recall or remember to bring documents, which would offload them of responsibilities.*

[P3]

### Current Workflow: Internal Organizational Policies, Procedures, and Culture

The findings from internal organizational policies, procedures, and culture suggest a need for stronger organizational support, more equitable access to medical information, and support for consistency in information management practices (Table 6). These are in relation to managing medical information within PDSD, but also in the context of Region Dalarna.

**Table 6.** Perspectives of organizational and procedural aspects categorized into the sociotechnical dimension of “internal organizational policies, procedures, and culture.”

Themes	Participants
Current documentation practices	
Existing routines	P1, P2, P3, P4, P5, P6, P7, P8, P9, P10, P12, P13, P14, P15, P16, P17, P18
Inconsistencies	P2, P5, P7, P9, P11, P18
System access limitation	
Inaccessible medication list platform	P3, P4, P5, P6, P8, P11, P12, P13, P14, P16, P17, P18
EHR^a^ access inequities	P5, P7, P9, P10, P11, P15, P17
Organizational support	
Organizational inaction	P1, P9, P15
Devalued	P11, P17

a EHR: electronic health record.

### Current Documentation Practices

In the category of current documentation practices, it was widely acknowledged that there are existing routines in place. The existing routines involved checking and updating information for every appointment, as well as reminding patients to bring their medication lists:

*We must always complete and review the patient’s medical history before starting treatment, in order to check whether anything has changed since their last visit.*

[P3]

*The routine is that, when booking an appointment, the receptionist tells patients to bring their medication list.*

[P9]

It also involved using a dedicated medical history section of EDR. Yet many participants noted inconsistencies in how these were applied, for example, documenting in progress notes or not updating medical information at all:

*We have a dedicated medical history section that patients fill out for every visit. Though some colleagues only record information in the progress notes instead of using it. That makes things difficult because you then have to dig through the entire record to find relevant information.*

[P5]

### System Access Limitations

An initiative mentioned by participants was “Förskrivningskollen*”* [51], an online platform enabling authorized users to view patients’ prescribed medications. However, it was described to be inaccessible due to technical issues or not provide enough information:

*There is a platform that we technically have access to […]. It is called ’Förskrivningskollen.’ While it shows prescriptions, it does not indicate what the patient is actually taking or the reasons behind those prescriptions.*

[P4]

The second barrier concerned EHR access inequities. While hospital-based or specialized dental clinics within PDSD have access to EHR systems, GDPs do not. Participants viewed this as an unjustified discrepancy:

*Dental practitioners working in hospital settings have access to EHR systems […] I asked around why we do not have the same access in GDPs, but no one could give me a clear answer.*

[P9]

### Organizational Support

Organizational support reflects how participants perceived the level of support provided for managing medical information in dental care, where organizational inaction refers to the lack of follow-through on identified issues, even after concerns were raised, and devalued captures the sense of not being prioritized by the broader organization:

*People often do not realize that dental treatments also depend on knowing about diseases and medications. I think many decision-makers do not understand our professional needs. We are frequently treated like the poor cousins of medical professionals, but what we do has a big impact. We do not want the information out of curiosity. We need it to work safely and effectively.*

[P11]

*Dentists need access to relevant medical information, and physicians also need greater knowledge of how diseases and medications affect oral health. More collaboration is needed, because it is ultimately about the patient.*

[P17]

### Current Workflow: External Rules, Regulations, and Pressures

The last sociotechnical dimension examined was external rules, regulations, and pressures. What can be derived from Table 7 is a need for clearer guidance and communication regarding applicable regulations in dental care regarding the subject matter.

**Table 7.** Overview of the categorization under the sociotechnical dimension of “external rules, regulations, and pressures.”

Themes	Participants
External regulations	
Awareness	P1, P2, P3, P5, P6, P10, P11, P14, P16
Standard practice	P4, P7, P8, P9, P12, P15, P17, P18
Patient consent	P5, P6, P11, P12, P14, P17, P18

### External Regulations

The results show that there is uncertainty about external regulations guiding the management of medical information in dental care. While many participants demonstrated awareness of existing regulations, several assumed that obtaining and documenting medical information is standard practice:

*I do not know of any rules that tell us explicitly to record medical history. I think it is obvious that we need to.*

[P9]

*National guidelines require us to document patients’ medical information.*

[P16]

Furthermore, the role of patient consent was perceived as crucial for retrieving medical information from both internal and external sources:

*Yes, the Patient Data Act states that dental practitioners and hygienists may access medical information, but only what is necessary for [providing] dental care. However, if we want to know patients’ medical history, we need their consent.*

[P14]

### Current Workflow: Conceptual Model

A UML activity diagram was developed to illustrate the current workflow for managing medical information in dental care based on the interview data (Multimedia Appendix 3). It outlines the key steps involved in acquiring, updating, and documenting medical information in the EDR system before proceeding with treatments. The diagram includes multiple actors, including receptionist, patient, dental practitioner, accompanying proxy, proxy, and medical care unit, each with defined responsibilities represented within separate partitions.

The workflow begins with the receptionist scheduling the appointment and reminding the patient to bring their medication list. Upon arrival, the dental practitioner checks the EDR to determine whether the patient is new or returning. For returning patients, the medical history section and progress notes are reviewed to assess whether updates are needed. If updates are required, or the patient is new, the dental practitioner checks if an accompanied proxy is present and able to provide the necessary information. If not, alternative steps may involve contacting another proxy or a relevant Medical Care Unit.

If no proxy is involved, the dental practitioner evaluates whether the patient can provide sufficient information via verbal recall, a medication list, or their online patient portal. If these sources are insufficient, attempts are made to contact a proxy or medical care unit, with the option to postpone the appointment.

Once the necessary information is obtained, it is documented in the EDR, either in the medical history section or progress notes. The dental practitioner may seek further medical consultation if needed. Once all relevant information is confirmed and documented, treatment can proceed.

### Potential Workflow: Conceptual Model

A UML activity diagram of a proposed workflow reflects potential improvements if medical information were made accessible in the EDR system (Multimedia Appendix 4). This revised workflow removes several steps previously required to manage information gaps and instead introduces a more linear progression toward care provision.

The dental practitioner begins by accessing and checking the medical information of the patient in their EDR system, with the receptionist not having to remind the patient to bring their medication list. Furthermore, there is no reliance on an accompanied proxy, proxy, and medical care unit to obtain medical information. Only if further clarification or consultancy is needed does the process escalate to contacting the medical care unit.

### Use Case Related to the Potential Workflow

**Figure 1.**
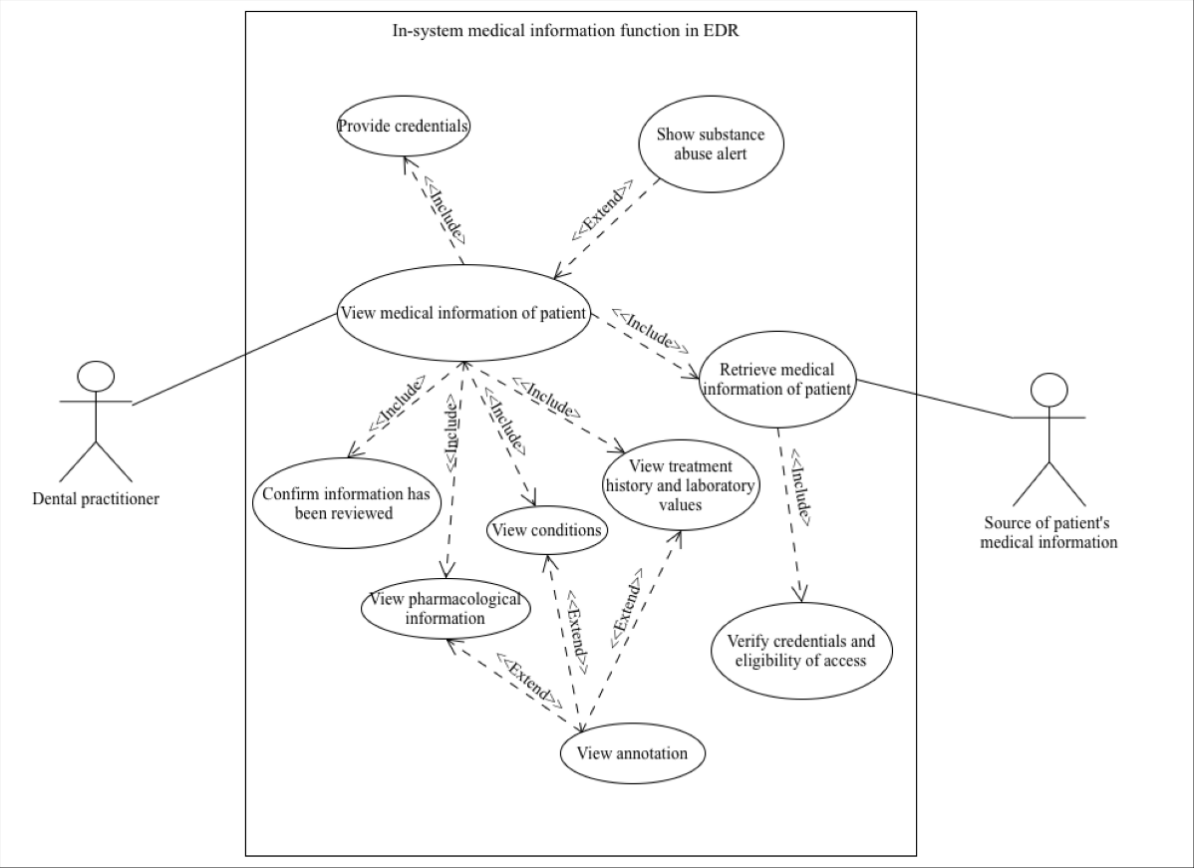
A use case with an electronic dental record system that involves providing access to medical information. EDR: electronic dental record.

A UML use case diagram (Figure 1) was developed to represent conceptual expectations and functional needs described by participants regarding an EDR system with access to medical information. This model also assumes that medical information is accessible via a regional database or NPÖ through NTjP, referred to as the source of the patient’s medical information in the diagram. It is important to emphasize that the use case diagram does not depict a user interface. Rather, it consolidates qualitative insights into conceptual recommendations. System-level specifications and exceptions are therefore not included.

Below follows the use case, with its corresponding process flow detailed in Table 8**:**

1. Use case name to view the medical information of a patient.
2. Description to describe the steps required for a dental practitioner to securely retrieve and review a patient’s medical information via an in-system function in the EDR system.
3. Alternative flow to ensure controlled data access, step 5 includes an alternative flow where, if the forwarded credentials are invalid or belong to a user without authorization, the data request is not processed.
4. Preconditions: the patient has given consent for a dental practitioner to access and view information, and the dental practitioner is directly involved in their care.
5. Postconditions: the patient’s medical information has been securely accessed and reviewed by the dental practitioner. The system has logged the confirmation of the reviewed information.

**Table 8.** Normal flow of events for accessing medical information.

Steps	Actors	Actions
1	Dental practitioner	Initiates action to view the patient’s medical information using an in-built function within the EDR^a^ system
2	In-system function	Prompts user authentication to verify access rights
3	Dental practitioner	Provides credentials
4	In-system function	Sends a data access request to the external system, including credentials
5	Source of patient’s medical information	Validates the access request and verifies that the credentials are valid and authorized to access medical information
6	Source of patient’s medical information	Retrieves and returns relevant medical data
7	In-system function	Displays relevant data in the conditions, pharmacological information, and treatment history and laboratory values sections
8	Dental practitioner	Reviews the provided information
9	Dental practitioner	Confirms that the information has been reviewed
10	In-system function	Logs the confirmation
11	Not applicable	End of use case

a EDR: electronic dental record.

### Sequential Flow of Accessing Medical Information in Potential Workflow

**Figure 2.**
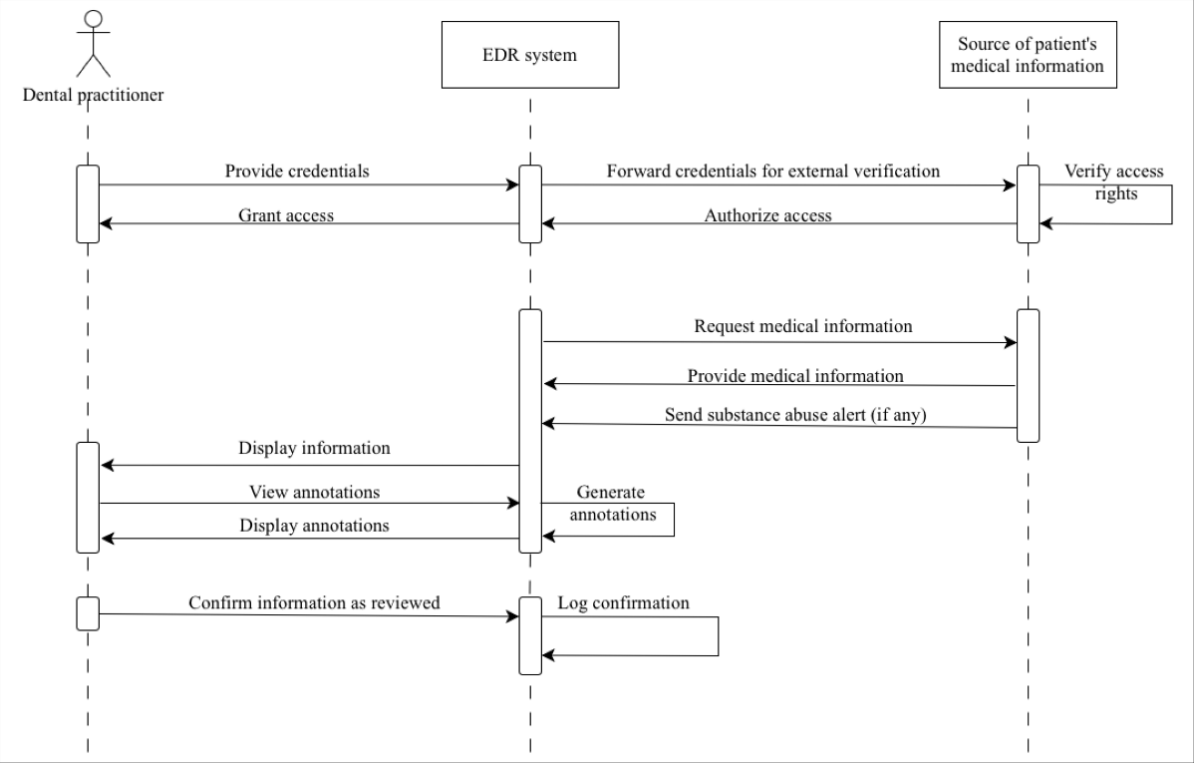
The sequence of processes in facilitating medical information. EDR: electronic dental record.

As shown in Figure 2, the UML sequence diagram depicts the interaction between the dental practitioner, the EDR system, and the source of patient’s medical information. After the dental practitioner provides credentials to the EDR system, a verification request is sent to the external source. Upon successful authorization, the EDR system sends a request and receives the patient’s medical information as a message. This may include substance abuse alerts, if applicable. The EDR system then displays the retrieved data to the dental practitioner, who may also choose to view system-generated annotations. Finally, the dental practitioner confirms that the information has been reviewed, which is logged in the system.

## Discussion

### Principal Results

#### Identified Medical Information Needs

Overall, the interviews consistently highlighted the need for accessible medical information to support safe, timely, and informed dental care provision. The findings predominantly aligned with what is reported in prior literature [3-5]. However, this study provided additional granularity regarding the specific medical information needs and their clinical relevance. Participants emphasized 4 main categories: conditions, pharmacological information, treatment history, and laboratory values.

Medical conditions such as cardiovascular disease, cancer, and psychiatric disorders were repeatedly cited due to their implications for procedural risk and treatment planning. Pharmacological needs centered on antiresorptive agents and anticoagulants, given their link to complications such as bleeding and osteonecrosis. Substance abuse history was also flagged as critical, particularly in the context of narcotic misuse. Allergies were a concern, especially penicillin allergy, due to its prescribing relevance. Moreover, relevant medical treatment history and laboratory values were also considered essential for planning and safety.

#### Identified Workflow Challenges

At GDPs in Dalarna, the current workflows are hindered by several challenges. These include a reliance on patients or third parties, which often leads to incomplete information gathering. Additionally, documentation workflows are fragmented, characterized by frequent interruptions and manual redundancy. The use of EDR for documenting medical information is inconsistent, and there is limited access to supportive information systems. These challenges represent a sociotechnical gap, where systems exist to store medical information but are not designed to meet the information needs or workflow constraints of the participants. In other words, the mere availability of medical information in EDR systems is insufficient, as usability, reliability, and contextual alignment were regarded as critical elements.

#### Workflow Barriers and Limitations

The findings underscore a misalignment between the current system design, organizational priorities, and clinical expectancy. Participants described relying heavily on patient self-reports, proxy input, or contacting external medical care units to obtain information. Patient self-reports were often unreliable, especially with elderly patients. Proxy or medical consultations introduced additional delays and communication gaps.

Despite the existence of routines, widespread inconsistencies in how medical histories are recorded were reported. Participants believed that having direct access to relevant medical information via the EDR would enhance treatment planning and reduce delays. However, they felt unsupported by the organization**,** citing failed efforts such as the online prescription check platform “Förskrivningskollen.”

The EDR system itself relies heavily on manual input, causing duplicated entries between the medical history section and progress notes. Manual data entry was perceived as burdensome, error-prone, and inefficient. Inconsistent documentation practices also impaired information reliability and visibility, requiring participants to spend additional time navigating and interpreting records.

There was also uncertainty about legal permissions for accessing medical records, despite the existence of the Integrated Health and Social Care Documentation Act (2022:913) and explicit clarifications from Swedish authorities [21,52]. This points to organizational and educational shortcomings rather than legal barriers. The forthcoming European health data space regulation may help address such ambiguity at the European Union level [53].

#### Proposed Improvements for Information Access

Enabling direct access to medical information via the EDR could streamline workflows by reducing reliance on manual data retrieval. This would improve time management, decision-making, and care coordination. A feasible path forward involves leveraging Sweden’s national interoperability infrastructure. By connecting EDR systems to NPÖ through NTjP, authorized dental professionals could retrieve relevant medical data across institutional boundaries [22-24].

#### Addressing Interoperability Levels

To implement this vision, interoperability must be achieved at four levels [11]: (1) technical, which must be supported through NTjP; (2) syntactic, which requires EDR systems to align with NTjP service contracts; (3) semantic, to provide a view-only use of external medical information, due to current dental records being largely unstructured; and (4) organizational, which requires commitment from vendors and stakeholders to align with interoperability frameworks and recognize dental professionals’ medical information access needs.

#### Toward a User-Centered Design Approach

Interface inefficiencies and inconsistent documentation stem not only from system limitations but also from design misalignment. Poorly designed systems, even with access to the right data, can obstruct rather than support care [3,13]. Given the high cognitive load and time pressure in dental care [54], systems should minimize user burden. Preferences expressed by participants underscore the need for user-centered EDR design regarding medical information.

#### Implications for Scalability and Policy Considerations

In highly urbanized or well-resourced Swedish regions, the proposed integration model would require significant adaptations to ensure scalability. These environments typically involve higher patient volumes, greater clinical complexity, and a more diverse ecosystem of EHR and EDR systems. To operate effectively under such conditions, the model must support real-time data synchronization, load balancing, and robust multisystem interoperability through standardized APIs, for example, applying the Health Level Seven Fast Healthcare Interoperability Resources standard and a modular system architecture. Larger regions may also necessitate granular, role-based access controls and comprehensive audit trails to uphold security and regulatory compliance. Scalable infrastructure, potentially leveraging cloud-based or hybrid deployment models, will be essential to accommodate periods with high system workload.

Beyond technical enhancements, successful large-scale implementation will depend on policy and infrastructural measures. These include enforcing national interoperability and terminology standards (eg, Fast Healthcare Interoperability Resources and Systematized Nomenclature of Medicine Clinical Terms), updating the Swedish Patient Data Act to explicitly enable cross-sector data sharing between dental and medical care while maintaining General Data Protection Regulation compliance, and allocating targeted funding to modernize legacy dental systems and support workforce training. Together, these considerations enhance the workflow’s scalability and support its potential application in settings that differ from the region studied.

#### Challenges of Integrating Artificial Intelligence–Driven Dental Imaging and Analysis Into EDR

Beyond the information needs described in this study, the rapidly expanding use of specialized dental software, such as artificial intelligence (AI)-driven digital smile design systems [55] and video-based smile analysis tools like Dynasmile [56], introduces additional challenges for integrating diverse data types into EDRs. These applications generate complex non-textual outputs, including high-resolution images, videos, and AI-derived esthetic or diagnostic metrics, which are increasingly used for treatment planning. Recent reviews of AI-based digital smile design indicate promising improvements in patient and clinician satisfaction, as well as esthetic outcomes [57]. Integrating such data into EDRs requires overcoming significant interoperability barriers. For instance, digital imaging data must comply with standards like Digital Imaging and Communications in Medicine to ensure consistent storage, retrieval, and portability across systems [58]. Similarly, AI-derived outputs (eg, landmark coordinates and esthetic scores) need semantic mapping to standardized clinical concepts to ensure meaningful linkage with diagnostic and treatment data. Furthermore, exchanging large multimedia files through NTjP raises practical considerations regarding storage, capacity, bandwidth, consent, and privacy compliance [59].

Acknowledging these challenges underscores the broader implications of our findings and highlights the need for future research on incorporating advanced dental imaging and AI-generated data into EDR workflows without compromising usability, interoperability, or patient confidentiality.

### Limitations

Certain limitations need to be declared. First, the sample consisted of 18 dental practitioners from PDSD-operated GDPs in Dalarna. Although the generalizability was limited due to the limited number of participants and the homogeneity of the sample, with most participants having fewer than five years of professional experience and working in similar clinical settings, thematic saturation was achieved in this study. Qualitative studies, besides providing an in-depth understanding of contextualized experience, may also support theoretical generalizability or, more appropriately for qualitative research, transferability when rich contextual information is provided [60]. This study supports the transferability of the findings to other contexts with similar characteristics. However, to strengthen the generalizability of these findings, future studies should include a broader and more diverse sample. A quantitative or mixed-methods study would also be valuable to validate and generalize the medical information needs identified here [61].

Future research could use quantitative methods to validate and prioritize the information needs identified in this study across a broader population of dental practitioners. Large-scale surveys using structured instruments (eg, Likert-scale items) could assess both the perceived importance and frequency of specific information needs, providing estimates of their prevalence and variability at regional or national levels. Additionally, a Delphi approach incorporating iterative rounds of quantitative scoring could facilitate structured expert consensus and systematic ranking of priorities.

### Conclusions

This study identified a clear need to improve access to medical information within EDR systems in GDPs in Dalarna. While medical information needs such as diagnoses, medications, and allergies are widely acknowledged in the literature, this study contributed detailed insights into the types of information most relevant to dental care. Current documentation practices were found to be fragmented, inefficient, and overly reliant on patients and third parties. Usability limitations in the EDR interface further hinder information access and consistency. Collectively, these issues reflect a sociotechnical misalignment between user needs and system capabilities. To address these gaps, the study proposes leveraging Sweden’s existing interoperability infrastructure, particularly by integrating EDR systems with NPÖ via NTjP. However, achieving this requires not only technical alignment but also organizational commitment, regulatory clarity, and a user-centered system design.

Future research should validate these findings at scale and explore system design and policy solutions to ensure that medical information can be securely, efficiently, and meaningfully integrated into information systems used in dental care.

## Supplementary material

10.2196/82691 Multimedia Appendix 1 Interview guide.

10.2196/82691 Multimedia Appendix 2 Consent form in Swedish and English.

10.2196/82691 Multimedia Appendix 3 Unified modeling language activity diagram of the current workflow in managing medical information.

10.2196/82691 Multimedia Appendix 4 Unified modeling language activity of an improved workflow if medical information were made accessible directly in the electronic dental record.

## Abbreviations

AI: artificial intelligence
EDR: electronic dental record
EHR: electronic health record
GDP: general dental practice
HIE: health information exchange
IEHR: integrated EHR-EDR
NPÖ: nationell patientöversikt
NTjP: nationell tjänsteplattform
PDSD: Public Dental Service Dalarna
UML: unified modeling language

## References

[1] Schmalz G, Brauer L, Haak R, Ziebolz D (2023). Evaluation of a concept to classify anamnesis-related risk of complications and oral diseases in patients attending the clinical course in dental education. BMC Oral Health.

[2] Harrel SK, Molinari J (2004). Aerosols and splatter in dentistry: a brief review of the literature and infection control implications. J Am Dent Assoc.

[3] Li S, Felix Gomez GG, Xu H, Rajapuri AS, Dixon BE, Thyvalikakath T (2024). Dentists’ information needs and opinions on accessing patient information via health information exchange: survey study. JMIR Form Res.

[4] Simon L, Obadan-Udoh E, Yansane AI (2019). Improving oral-systemic healthcare through the interoperability of electronic medical and dental records: an exploratory study. Appl Clin Inform.

[5] Alanazi A, Alghamdi G, Aldosari B (2023). Informational needs for dental-oriented electronic health records from dentists’ perspectives. Healthcare (Basel).

[6] Lisiecka MZ (2025). Allergic reactions in dental practice: classification of medicines, mechanisms of action, and clinical manifestations. Clin Rev Allergy Immunol.

[7] Adibi S, Li M, Salazar N (2020). Medical and dental electronic health record reporting discrepancies in integrated patient care. JDR Clin Trans Res.

[8] Charangowda BK (2010). Dental records: an overview. J Forensic Dent Sci.

[9] Evans RS (2016). Electronic health records: then, now, and in the future. Yearb Med Inform.

[10] Heid DW, Chasteen J, Forrey AW (2002). The electronic oral health record. J Contemp Dent Pract.

[11] Lehne M, Sass J, Essenwanger A, Schepers J, Thun S (2019). Why digital medicine depends on interoperability. NPJ Digit Med.

[12] Holmgren AJ, Adler-Milstein J (2017). Health information exchange in us hospitals: the current landscape and a path to improved information sharing. J Hosp Med.

[13] Taylor HL, Apathy NC, Vest JR (2020). Health information exchange use during dental visits. AMIA Annu Symp Proc.

[14] Li S, Rajapuri AS, Felix Gomez GG, Schleyer T, Mendonca EA, Thyvalikakath TP (2022). How do dental clinicians obtain up-to-date patient medical histories? modeling strengths, drawbacks, and proposals for improvements. Front Digit Health.

[15] Atchison KA, Weintraub JA, Rozier RG (2018). Bridging the dental-medical divide: case studies integrating oral health care and primary health care. J Am Dent Assoc.

[16] (2019). About the Swedish healthcare system. Socialstyrelsen.

[17] (2022). Uppföljning Vision e-hälsa 2025 – Rapport avseende år 2021 (Report in Swedish). https://www.ehalsomyndigheten.se/globalassets/ehm/3_om-oss/rapporter/uppfoljning-vision-e-halsa-2025-rapport-avseende-2021.pdf.

[18] (2023). Follow-Up Vision for eHealth 2025 - Report on the Year 2022. https://www.ehalsomyndigheten.se/siteassets/ehm/3_om-ehalsomyndigheten/rapporter/rapporter_regeringsuppdrag/follow-up-vision-for-ehealth-2025-report-on-the-year-2022.pdf.

[19] Swedish Institute Swedish government. Power for the people! This is how Sweden is governed.

[20] Nilsson P, Stjernquist A, Janlöv N (2016). Fragmented health and social care in Sweden - a theoretical framework that describes the disparate needs for coordination for different patient and user groups. Int J Integr Care.

[21] (2022). The Government Offices’ legal databases [Web page in Swedish]. Regeringskansliet.

[22] (2022). Sammanhållen journalföring – Möjligheter till digital informationsförsörjning på hälsodataområdet. https://www.ehalsomyndigheten.se/globalassets/ehm/3_om-oss/rapporter/slutrapportering-av-uppdraget-att-foresla-hur-sammanhallen-journalforing-kan-nyttjas-i-storre-utstrackning.pdf.

[23] Nationell patientöversikt – NPö [Web page in Swedish]. Inera.

[24] Nationella tjänsteplattformen. Inera.

[25] (2017). Långt kvar till gemensamt IT-system [Web page in Swedish]. Tandläkartidningen.

[26] Atchison KA, Weintraub JA (2017). Integrating oral health and primary care in the changing health care landscape. N C Med J.

[27] MacNeil RLM, Hilario H, Ryan MM, Glurich I, Nycz GR, Acharya A (2020). The case for integrated oral and primary medical health care delivery: marshfield clinic health system. J Dent Educ.

[28] Shimpi N, Glurich I, Panny A, Acharya A (2019). Knowledgeability, attitude, and practice behaviors of primary care providers toward managing patients’ oral health care in medical practice: Wisconsin statewide survey. J Am Dent Assoc.

[29] Harnagea H, Couturier Y, Shrivastava R (2017). Barriers and facilitators in the integration of oral health into primary care: a scoping review. BMJ Open.

[30] (2023). Unika siffror: här är tandläkarbristen värst. Tandläkartidningen.

[31] Dalarna. Tillväxtverket.

[32] Janto M, Iurcov R, Daina CM (2022). Oral health among elderly, impact on life quality, access of elderly patients to oral health services and methods to improve oral health: a narrative review. J Pers Med.

[33] Alami H, Lehoux P, Gagnon MP, Fortin JP, Fleet R, Ag Ahmed MA (2020). Rethinking the electronic health record through the quadruple aim: time to align its value with the health system. BMC Med Inform Decis Mak.

[34] Baxter G, Sommerville I (2011). Socio-technical systems: from design methods to systems engineering. Interact Comput.

[35] Hunter DJ, McCallum J, Howes D (2018). Defining exploratory-descriptive qualitative (EDQ) research and considering its application to healthcare. Proceedings of the 6th Annual Worldwide Nursing Conference 2018.

[36] Tandvård genom hela livet [Web page in Swedish]. Region Dalarna.

[37] Ordell S, Unell L, Söderfeldt B (2006). An analysis of present dental professions in Sweden. Swed Dent J.

[38] Palinkas LA, Horwitz SM, Green CA, Wisdom JP, Duan N, Hoagwood K (2015). Purposeful sampling for qualitative data collection and analysis in mixed method implementation research. Adm Policy Ment Health.

[39] DeJonckheere M, Vaughn LM (2019). Semistructured interviewing in primary care research: a balance of relationship and rigour. Fam Med Community Health.

[40] Sittig DF, Singh H (2010). A new sociotechnical model for studying health information technology in complex adaptive healthcare systems. Qual Saf Health Care.

[41] Saunders B, Sim J, Kingstone T (2018). Saturation in qualitative research: exploring its conceptualization and operationalization. Qual Quant.

[42] Vaismoradi M, Turunen H, Bondas T (2013). Content analysis and thematic analysis: Implications for conducting a qualitative descriptive study. Nurs Health Sci.

[43] Neale J (2016). Iterative categorization (IC): a systematic technique for analysing qualitative data. Addiction.

[44] Ciccozzi F, Malavolta I, Selic B (2019). Execution of UML models: a systematic review of research and practice. Softw Syst Model.

[45] Aggarwal V (2002). The application of the unified modeling language in object-oriented analysis of healthcare information systems. J Med Syst.

[46] Lag om etikprövning av forskning som avser människor [Web page in Swedish]. SVERIGES RIKSDAG.

[47] What the Act says [Web page in Swedish]. Etikprövningsmyndigheten.

[48] European Parliament and Council of the European Union (2016). Regulation (EU) 2016/679 of the European Parliament and of the Council of 27 April 2016 on the protection of natural persons with regard to the processing of personal data and on the free movement of such data (General Data Protection Regulation). Off J Eur Union.

[49] Association WM (2013). World Medical Association Declaration of Helsinki: ethical principles for medical research involving human subjects. JAMA.

[50] Om oss [Web page in Swedish]. Carestream dental.

[51] Förskrivningskollen [Web page in Swedish]. E-hälsomyndigheten.

[52] (2019). Kartläggning av hinder för samverkan mellan tandvård och hälso- och sjukvård – slutrapport 2019 [Web page in Swedish]. Socialstyrelsen.

[53] European Health Data Space Regulation (EHDS). European Commission.

[54] Pontes CC, Stanley K, Molayem S (2024). Understanding the dental profession’s stress burden: prevalence and implications. Compend Contin Educ Dent.

[55] Khaled Ali GA, Abdelbadei KA, Essam M, Ali GAK Smile.AI: a deep learning system for digital smile design.

[56] Chen K, Qiu L, Xie X, Bai Y (2025). Dynasmile: video-based smile analysis software in orthodontics. SoftwareX.

[57] Saini RS, Kaur K, Gurumurthy V (2025). Impact of artificial intelligence-based digital smile design on patient and clinician satisfaction and facial esthetic outcomes: a systematic review and meta-analysis. Digit Health.

[58] Farman AG (2002). Use and implication of the DICOM standard in dentistry. Dent Clin North Am.

[59] Liu TY, Lee KH, Mukundan A, Karmakar R, Dhiman H, Wang HC (2025). AI in dentistry: innovations, ethical considerations, and integration barriers. Bioengineering (Basel).

[60] Carminati L (2018). Generalizability in qualitative research: a tale of two traditions. Qual Health Res.

[61] Polit DF, Beck CT (2010). Generalization in quantitative and qualitative research: myths and strategies. Int J Nurs Stud.

