# Supplementary material for: Exploring Medical Information Needs and Accessibility in Swedish Dental Care by Analysis of Documentation Workflows and Electronic Dental Records in Dalarna: Sociotechnical Qualitative Study
Source: JMIR Hum Factors. 2026 Jan 29;13:e82691. doi: 10.2196/82691 (PMC12854659; doi:10.2196/82691)
Supplement: Multimedia Appendix 1 [file humanfactors-v13-e82691-s001.pdf]

Multimedia Appendix 1. Interview Guide

(In English)

Introduction

- Ask if the participant wants the interview to be held in English or Swedish
- Briefly introduce myself and the purpose of the study.
    - Provide an overview of the study's purpose: *to explore the information needs of patients among dental practitioners in Dalarna and assess challenges related to accessing patient data from the medical domain*.
    - *Explain the term medical information in regard to dental and medical care*.
- Explain the interview format and the expected duration (30 minutes).
- Explain the confidentiality and anonymization of the participants and responses.
- Seek informed consent (written and verbal) and clarify rights to withdraw.

Section 1: Background Information

1. Can you describe your current role in dentistry?
2. Age and sex?
3. Did you receive your training in Sweden or abroad?
4. How long have you been working in this field?
5. How long have you been working in this field in Dalarna?
6. Which municipality is your practice located in?
7. What kinds of patients do you typically treat (elderly, children, medically complex patients, etc.)?
8. What EDR system are you currently using?
9. In the Electronic Dental Record system, do you generally document patient information in free text or in standardized format (e.g. coding system, standardized vocabulary, terminology)?
10. Do you consider medical information of patients necessary for your clinical workflow?

Section 2: Clinical Content

11. What types of medical information of patients do you consider essential to have for your clinical workflow? Why?
12. Are there specific types of medical information of patients that you believe should be more accessible to in the existing system you are using? Why?

Section 3: People

13. How do you currently obtain patients' medical history?
14. Are there specific challenges you face in obtaining patients' medical information?
15. How often do you need to contact a medical/healthcare professional to clarify a patient's medical history? If so, is there any specific medical profession, discipline or institution you contact?
16. Do you have an example of encountering difficulties in communicating with healthcare/medical professionals about patient information?

Section 4: Human-Computer Interface

17. Do you find your current system user-friendly, or do you experience challenges when retrieving or inputting medical information?
18. Do you document medical information in free text or by using standardized coding systems, and are there any problems you see using either method?
19. How would you prefer to have patients' medical information made accessible in your current system? Would you prefer to have it integrated into the current system or separately?

Section 5: Workflow and Communication

20. Describe your current workflow for obtaining patients' medical information, including how you retrieve and update this information in the existing system.
21. Are there instances where missing medical information of patients has impacted your clinical workflow, as in decision-making, treatment planning or care delivery? How?
22. How would having better access to patients' medical information improve the processes of treatment planning and decision-making in your practice?

Section 6: Internal Organizational Policies and Procedures

23. Are there any existing policies in your practice regarding medical history documentation and retrieval?
24. Are there any organizational changes that you think could improve access to medical information of patients?
25. Are you aware of any regional initiatives in facilitating patients' medical information to dental care?

Section 7: External Rules, Regulations and Pressure

26. Are there any external regulations or rules that govern how you should document medical information?

Section 8: Follow-up question

27. Is there anything more you like to add regarding the topic?

(In Swedish)

Introduktion

- Fråga först om deltagaren önskar att ha intervjun på engelska eller svenska.
- En kort presentation av mig själv och syftet med studien.
    - Ge en översikt över studiens syfte: att undersöka behovet av anamnes och patientuppgifter bland tandvårdspersonal i Dalarna samt de utmaningar som är förknippade med att få åtkomst till medicinska uppgifter från hälso- och sjukvården.
    - Klargör begreppet medicinska uppgifter i samband med tand- och sjukvård..
- Förklara intervjuns upplägg och den beräknade tidsåtgången (30 minuter)
- Information om sekretess och anonymitet.
- Inhämta samtycke (skriftligt och muntligt) och informera om rätten att avbryta sin medverkan.

Sektion 1: Klinisk bakgrundsinformation

1. Kan du beskriva din nuvarande roll inom tandvården?
2. Ålder och kön?
3. Genomförde du din utbildning i Sverige eller utomlands?
4. Hur länge har du arbetat inom detta yrkesområde?
5. Hur länge har du arbetat inom detta yrkesområde i Dalarna?
6. I vilken kommun är din klinik belägen i?
7. Vilka typer av patienter behandlar du vanligtvis (äldre, barn, multisjuka patienter etc.)?
8. Vilket journalsystem använder du?
9. I tandvårdsjournalen, dokumenterar du generellt patientuppgifter i fritext eller i standardiserad format (t.ex. kodningssystem, terminologier)?
10. Anser du att medicinska uppgifter från patienter är viktiga för ditt kliniska arbete?

Sektion 2: Informationshantering

11. Vilka typer av patienters medicinska uppgifter anser du vara viktigt för ditt kliniska arbete? Varför?
12. Finns det specifika typer av medicinska uppgifter som du anser borde vara mer tillgänglig inom tandvården i den nuvarande journalsystemet du använder? Varför?

Sektion 3: Samverkan med individer

13. Hur får du i dagsläget tillgång till patienters medicinska uppgifter?
14. Finns det specifika utmaningar du stöter på vid insamling av medicinska uppgifter från patienter?
15. Hur ofta behöver du kontakta vårdpersonal inom hälso-och sjukvården för att klargöra patienters medicinska historia? I så fall, kontaktar du någon specifik profession, instans, eller specialitet?
16. Har du något exempel på när du har stött på svårigheter i kommunikationen med vårdpersonal gällande patientuppgifter?

Sektion 4: Människa-datorinteraktion

17. Upplever du att ditt nuvarande journalsystem är användarvänligt, eller stöter du på utmaningar vid inmatning eller tillgång till patienters medicinska uppgifter?

18. Dokumenterar du medicinska uppgifter i fritext eller genom standardiserade kodningssystem, och anser du att det finns problem i den metod som används?
19. Om medicinska uppgifter gjordes tillgänglig för dig i den nuvarande journalsystemet, hur skulle du föredra att få åtkomst till det? Skulle du vilja ha det integrerat i det nuvarande systemet eller separat?

Sektion 5: Arbetsflöde och kommunikation

20. Beskriv ditt nuvarande arbetsflöde för att inhämta patienters medicinska uppgifter, inklusive hur du får åtkomst och uppdaterar denna information i tandvårdsjournalen.
21. Har det funnits tillfällen där avsaknaden av medicinska uppgifter har påverkat dina kliniska beslut, behandlingsplaner eller vården i sig? Hur?
22. Hur skulle bättre tillgång till medicinska uppgifter påverka din behandlingsplanering och kliniska beslutsfattanden i vardagen?

Sektion 6: Interna policy och rutiner

23. Finns det några befintliga bestämmelser på din arbetsplats som reglerar dokumentation och åtkomst av patienters medicinska uppgifter?
24. Finns det några organisatoriska förändringar som du anser skulle kunna förbättra tillgången till patienters medicinska uppgifter?
25. Är du medveten om några regionala initiativ som syftar till att underlätta tillgången till patientuppgifter eller medicinska uppgifter för tandvården?

Sektion 7: Externa regler, föreskrifter och påtryckningar

26. Finns det externa regler eller krav som ställs i hur du ska dokumentera medicinska uppgifter i tandvårdsjournalen?

Sektion 8: Övrigt:

27. Finns det något annat du vill tillägga gällande ämnet som diskuterats?
