# Supplementary material for: Exploring Medical Information Needs and Accessibility in Swedish Dental Care by Analysis of Documentation Workflows and Electronic Dental Records in Dalarna: Sociotechnical Qualitative Study
Source: JMIR Hum Factors. 2026 Jan 29;13:e82691. doi: 10.2196/82691 (PMC12854659; doi:10.2196/82691)
Supplement: Multimedia Appendix 2 [file humanfactors-v13-e82691-s002.pdf]

Multimedia Appendix 2. Information and Consent Form

(In English)

Consent Form

Project Title: Exploring the Accessibility and Needs of Medical Information in Dental Care: A Qualitative Interview-Based Study in Dalarna, Sweden

This study is part of a degree project in Health Informatics at Karolinska Institutet, Sweden

Principal Instigator:
Sahid Hasan Rahim, Master's Student, Joint Master's Programme in Health Informatics, Karolinska Institutet
sahid.hasan.rahim@stud.ki.se

Main supervisor:
Stefano Bonacina, MSc, PhD,
Assistant Senior Lecturer,
Department of learning, informatics, management, and ethics,
Karolinska Institutet
stefano.bonacina@ki.se

1. Introduction

You are invited to participate in an interview for a research study exploring the accessibility and needs of medical information amongst dental practitioners in Dalarna, Sweden. The purpose of this study is to understand the needs and challenges dental practitioners face in obtaining accurate medical information about patients that is relevant to their practice. Such information may be more accurately documented in Electronic Health Record (EHR) systems at medical care units, which could be beneficial if made available to dental practitioners through their Electronic Dental Record (EDR) systems. Having accurate medical information available is particularly important as dental practitioners may need to care for medically complex patients or patients having traveled from distant locations with no record of their medical history in the EDR system. However, before proposing any solutions, it is essential to first gain a comprehensive understanding of the dental workflow and, if present, the specific needs in relation to this topic.

2. About the Interview

Upon agreeing to participate, you will partake in a semi-structured interview lasting about 30 minutes. The questions will focus on your professional experience, the current workflow for retrieving, obtaining, and storing medical information, and the needs and challenges related to these processes within your current EDR system. Your responses will be recorded and transcribed using a computer application. To minimize bias, no reimbursement or compensation will be provided for participation in this study.

3. Use of Interview Responses

The interview responses will be used for writing a thesis on the topic, which may be published in scientific journals in the future. The findings may lead to recommendations for improving data accessibility in dental care in Dalarna, with the prospect of enhancing patient care.

4. Your Rights as a Participant

Participation is voluntary, and you can withdraw at any time without providing a reason. You may refuse to answer any question you do not feel comfortable with. Additionally, you have the right to access the data collected about you, request corrections, or ask for the data to be deleted at any time before the study is published.

5. Confidentiality and Data Protection

Your responses will be anonymized, ensuring that no personally identifiable information, such as your name, workplace, or other identifying details, will be included in the final analysis. This study adheres to the EU General Data Protection Regulation (GDPR) and the Swedish Data Protection Act (2018:218). Only the research team will have access to the raw data. Any direct quotes used in the study will be presented anonymously. All recordings, transcripts, and data will be securely stored and deleted once the study is completed and published.

4. Contact Information for Further Inquiries

Should you seek further information about this research study or about your participation, please contact Sahid Hasan Rahim at sahid.hasan.rahim@stud.ki.se.

4. Consent Statement

By signing this form, you confirm that:

- You have read and understood the information provided.
- You voluntarily agree to participate in this study.
- You understand that your responses will be anonymized and kept confidential.
- You are aware that you can withdraw at any time without any consequences.

Place and date	Name and signature of participant

Place and date	Name and signature of instigator

(In Swedish)

Samtyckesformulär

Projekttitel: "Exploring the Accessibility and Needs of Medical Information in Dental Care: A Qualitative Interview-Based Study in Dalarna, Sweden"

Studien är en del av ett examensarbete i hälsoinformatik på Karolinska institutet.

Huvudanstiftare:
Sahid Rahim, mastersstudent,
Mastersprogrammet i hälsinformatik,
Karolinska Institutet
sahid.hasan.rahim@stud.ki.se

Handledare:
Stefano Bonacina, MSc, PhD, biträdande lektor,
Institutionen för lärande, informatik,
management och etik, Karolinska Instituet
stefano.bonacina@ki.se

1. Introduktion

Du är inbjuden till att delta i en intervju för en forskningsstudie som undersöker informationsbehoven hos tandvårdspersonalen i Dalarna, Sverige. Syftet med denna studie är att förstå de behov och utmaningar som tandvårdspersonal står inför när det gäller att erhålla korrekt medicinsk information om patienter som är relevanta för deras yrkesutövning. Sådan information kan vara mer korrekt dokumenterade i hälso- och sjukvårdens journalsystem, vilket skulle kunna vara fördelaktigt för tandvårdspersonal att ha åtkomst till genom deras tandvårdsjournalsystem. Att ha korrekt medicinsk information tillgänglig är särskilt viktigt då tandvårdspersonal kan behöva ta hand om multisjuka patienter eller patienter som har rest från avlägsna platser där anamnesuppgifter i tandvårdsjournalen fattas. Innan man kan föreslå några lösningar är det dock nödvändigt att först få en omfattande förståelse för tandvårdspersonalens arbetsflöde och de specifika behoven som finns i relation till detta område.

2. Om intervjun

Om du samtycker kommer du att delta i en semi-strukturerad intervju som varar i cirka 30 minuter. Frågorna kommer att fokusera på din yrkeserfarenhet, det nuvarande arbetsflödet för att hämta, erhålla och lagra medicinsk information, samt de behov och utmaningar som är relaterade till tandvårdsjournalen. Dina svar kommer att spelas in och transkriberas med hjälp av programvara. För att minimera faktorer som kan påverka studiens utfall kommer ingen ersättning eller kompensation att delges.

3. Om dina svar

Dina svar från intervjun kommer att användas för att skriva en avhandling om ämnet, vilket kan komma att publiceras i vetenskapliga tidskrifter i framtiden. Resultaten kan leda till rekommendationer för att förbättra dataåtkomst inom tandvården i Dalarna, med målet att förbättra patientomhändertagandet.

4. Dina rättigheter som deltagare

Deltagande är frivilligt, och du kan när som helst avbryta din medverkan utan att ange någon orsak. Du har rätt att vägra att svara på någon fråga som du inte känner dig bekväm med. Dessutom har du rätt att få tillgång till de uppgifter som samlats in om dig, begära korrigeringar eller be om att dina uppgifter raderas när som helst innan studien publiceras.

5. Sekretess och dataskydd

Dina svar kommer att anonymiseras, vilket innebär att inga identifierbara personuppgifter, såsom namn, arbetsplats samt övriga identifierande uppgifter, kommer att ingå i den slutliga analysen. Denna studie följer EU:s allmänna dataskyddsförordning (GDPR) samt den svenska dataskyddslagen (2018:218). Endast ansvariga för studien kommer att ha tillgång till all data. Eventuella citat som används i studien kommer att presenteras anonymt. All inspelning, transkribering och data kommer att lagras på ett säkert sätt och raderas när studien är avslutad och publicerad.

6. Kontaktinformation för ytterligare frågor

Om du har några frågor om denna forskningsstudie eller om ditt deltagande, vänligen kontakta Sahid Hasan Rahim på sahid.hasan.rahim@stud.ki.se.

7. Samtyckesförklaring

Genom att signera blanketten bekräftar du att:

- Du har läst och förstått informationen ovan.
- Du frivilligt samtycker till att delta i denna studie.
- Du förstår att dina svar kommer att vara anonyma och hållas enligt gällande sekretesslagar och föreskrifter.
- Du är medveten om att du kan avbryta ditt medverkande när som helst utan någon påverkan.

Ort och datum	Namn och signatur av deltagare

Ort och datum	Namn och signatur av huvudanstiftare
