# Supplementary figures and images for: Exploring Medical Information Needs and Accessibility in Swedish Dental Care by Analysis of Documentation Workflows and Electronic Dental Records in Dalarna: Sociotechnical Qualitative Study
Source: JMIR Hum Factors. 2026 Jan 29;13:e82691. doi: 10.2196/82691 (PMC12854659; doi:10.2196/82691)

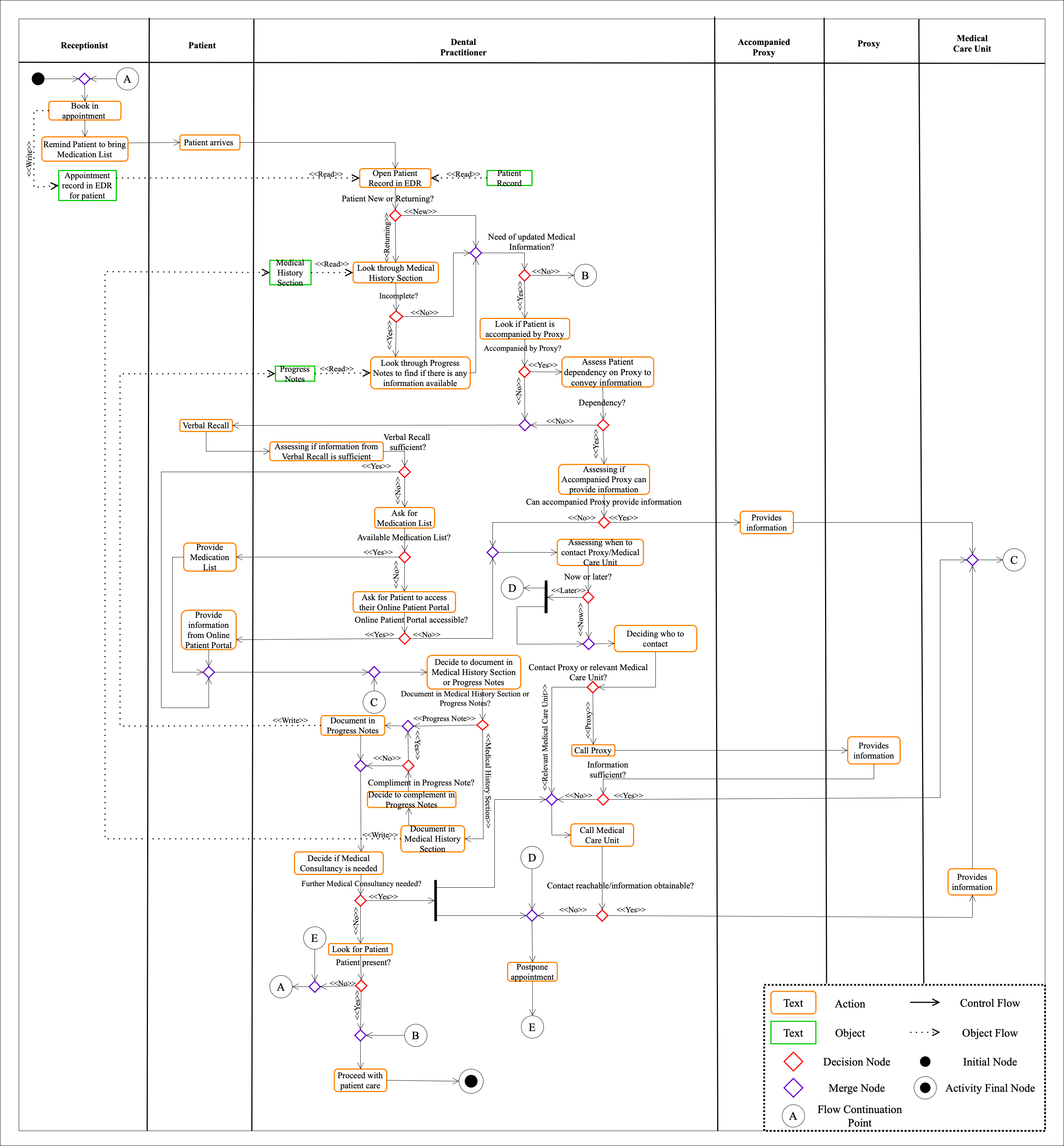

Supplement: Multimedia Appendix 3 [file humanfactors-v13-e82691-s003.png]

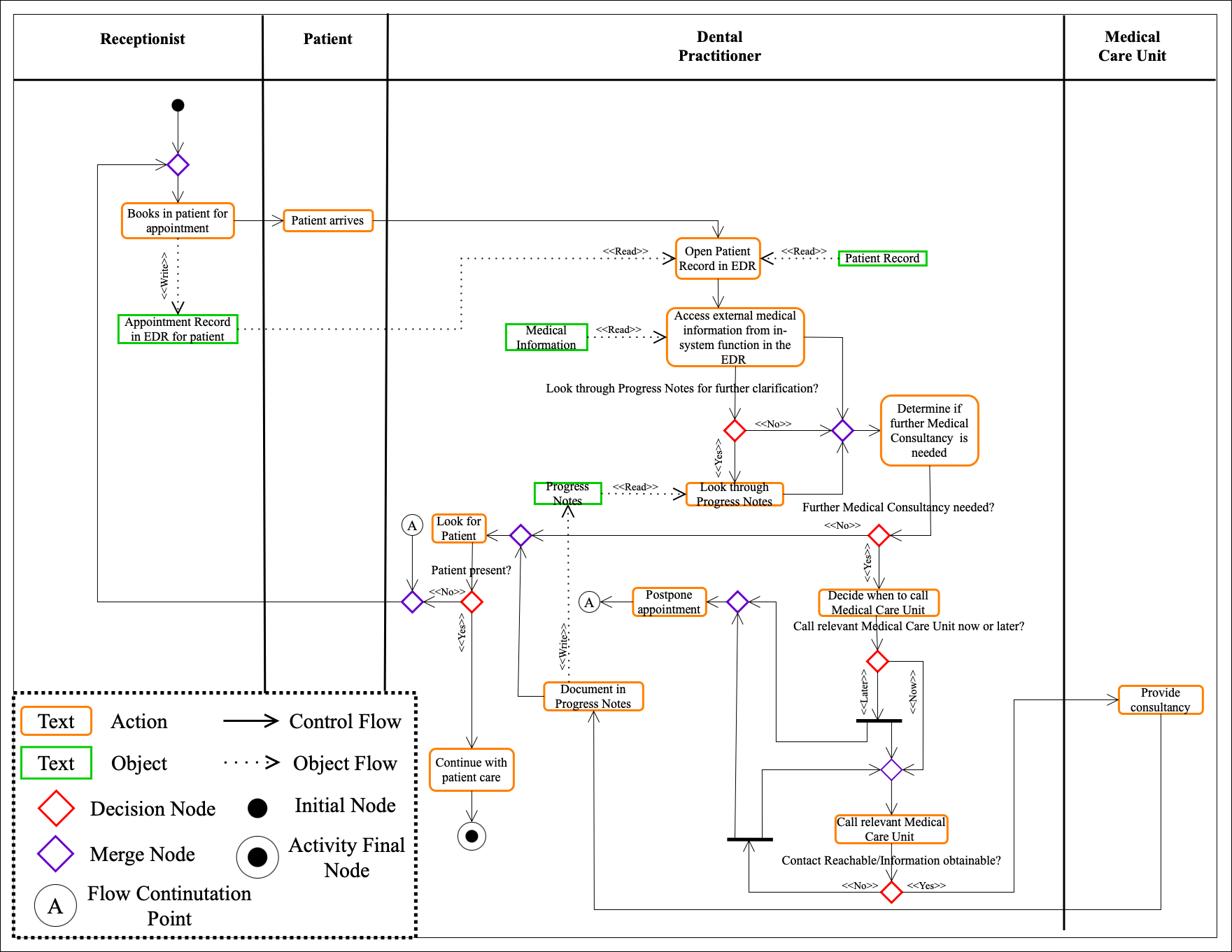

Supplement: Multimedia Appendix 4 [file humanfactors-v13-e82691-s004.png]
